# Antibiotics- and Heavy Metals-Based Titanium Alloy Surface Modifications for Local Prosthetic Joint Infections

**DOI:** 10.3390/antibiotics10101270

**Published:** 2021-10-19

**Authors:** Jaime Esteban, María Vallet-Regí, John J. Aguilera-Correa

**Affiliations:** 1 Clinical Microbiology Department, Jiménez Díaz Foundation Health Research Institute, Autonomous University of Madrid, Av. Reyes Católicos 2, 28040 Madrid, Spain; 2 Networking Research Centre on Infectious Diseases (CIBER-ID), 28029 Madrid, Spain; 3 Department of Chemistry in Pharmaceutical Sciences, Research Institute Hospital 12 de Octubre (i+12), School of Pharmacy, Complutense University of Madrid, Pza. Ramón y Cajal s/n, 28040 Madrid, Spain; vallet@ucm.es; 4 Networking Research Centre on Bioengineering, Biomaterials and Nanomedicine (CIBER-BBN), 28029 Madrid, Spain

**Keywords:** prosthetic joint infection, local prevention

## Abstract

Prosthetic joint infection (PJI) is the second most common cause of arthroplasty failure. Though infrequent, it is one of the most devastating complications since it is associated with great personal cost for the patient and a high economic burden for health systems. Due to the high number of patients that will eventually receive a prosthesis, PJI incidence is increasing exponentially. As these infections are provoked by microorganisms, mainly bacteria, and as such can develop a biofilm, which is in turn resistant to both antibiotics and the immune system, prevention is the ideal approach. However, conventional preventative strategies seem to have reached their limit. Novel prevention strategies fall within two broad categories: (1) antibiotic- and (2) heavy metal-based surface modifications of titanium alloy prostheses. This review examines research on the most relevant titanium alloy surface modifications that use antibiotics to locally prevent primary PJI.

## 1. Introduction

The use of arthroplasty makes it possible to replace a natural joint with artificial material or a joint prosthesis. Although, arthroplasty is highly effective and has improved the quality of life of millions of patients [1], implant-related complications can appear during the lifetime of patients [2]. One of the most important complications is prosthetic joint infection (PJI), although others may occur. This is probably the most devastating complication due to the high morbidity, mortality, and costs associated with PJI. The mean cost per patient with knee PJI of is USD 52,555 (EUR 40,542), with a range of between USD 24,980 (EUR 19,270.80) for patients with early PJI, and USD 78,111 (EUR 60,257) for those with late PJI [3]. Incidence varies from country to country, between 0.5–2%. Thus, PJI incidence is ranged between 1 and 2% in the United States, and between 0.6% and 0.72% in Nordic countries [4,5]. It is important to know this incidence could be higher in patients undergoing a primary arthroplasty with a history of a PJI in another joint showing up to a three-fold higher risk of PJI [6]. Currently, the 5-year mortality rate associated with PJI is greater than that of breast cancer, melanoma, and Hodgkin’s lymphoma [7].

The aim of this work is to review research on the most relevant titanium alloy surface modifications that use antibiotics to locally prevent primary PJI.

## 2. Etiopathology

Staphylococci, including *Staphylococcus aureus* (30–40%) and different species of coagulase-negative staphylococci (27–43%), among which *S. epidermidis* predominates, are the most common etiological agents associated with PJI [8,9,10,11,12]. Among Gram-negative bacteria (3–9%) [13], enterobacteria and non-fermenting Gram-negative bacilli stand out. However, there could be differences in these patterns according to the characteristics of the infection [9] or the affected joint [14,15]. Polymicrobial infections, or those caused by more than one microorganism, may occur in 10–35% of cases [2,13]. *Enterococcus* species, *Staphylococcus,* and various Gram-negative bacilli such as Enterobacteriaceae and *Pseudomonas aeruginosa* are often associated with these infections.

A problem of growing importance associated with bacterial infections is antibiotic resistance [16]. According to the Centers for Disease Control, approximately 2.8 million antibiotic-resistant bacterial infections take place in the United States and provoke more than 35,000 deaths every year [17]. The main bacteria related to this antibiotic resistance are (as declared by the WHO) *Acinetobacter baumannii*, *P. aeruginosa*, enterobacteria (e.g., *Klebsiella pneumoniae* and *Enterobacter cloacae*), *Enterococcus faecium*, *S. aureus*, *Helicobacter pylori*, *Campylobacter* spp., *Salmonella* spp., *Neisseria gonorrhoeae*, *Streptococcus pneumoniae*, *Haemophilus influenzae*, and *Shigella* spp. [16,18]. As can be seen, many of the listed bacteria are causative agents of PJI, e.g., *S. aureus*, *P. aeruginosa*, *K. pneumonia*, *E. cloacae*, and *E. faecium*, and for that, the antibiotic resistance is also an emerging threat for PJI and must be taken into account in the development of any preventive treatment against them.

One of the most important characteristics in all the aspects of PJI is the ability of microorganisms to form biofilms. A biofilm is a conglomerate of microbial cells of at least one species that is irreversibly attached or not on a surface or an interface, and embedded in a self-produced matrix of polymeric extracellular substances [19], where numerous complex sociomicrobiological interactions prevail [20,21,22]. It is estimated that at least 80% of chronic infections are directly related to the ability of the causative microorganism to develop a biofilm, likely including 100% of all implant-related infections [23,24]. Biofilm formation involves at least three different stages:

(1) Attachment. Microorganisms come into contact with the surface, a process that is at least partly stochastic, driven by physical and chemical forces [25,26,27]. Furthermore, host proteins rapidly coat the surface of medical devices, facilitating specific adhesion mediated by microbial surface components recognizing adhesive matrix molecules (MSCRAMMs), which are part of the surface of many bacteria, e.g., *Staphylococcus* spp. [28,29].
(2) Maturation is characterized by intercellular aggregation coupled to a variety of molecules such as proteins or, usually, exopolysaccharides of a polysaccharide nature, and structuring forces that rearrange the biofilm into three-dimensional structures of variable morphology depending on the species and with microchannels within them [28]. During this stage, one of the most important processes is the production of the exopolysaccharide matrix, whose composition is characteristic of each species, and even of each strain [28,29,30,31]. At this stage, the relatively simple structure that the pre-biofilm acquired in irreversible adhesion takes on a much more structurally complex three-dimensional organization [32]. The nutritional gradient inside the biofilm gives rise to a variety of cells with metabolic differences, including starved cells, dormant cells, viable non-cultivable cells, “persister” cells, and dead cells [27,33].
(3) Dispersal. This is the process by which mature biofilm cells disperse to adjacent areas passively or actively [23,27]. Through this stage, the infection spreads to adjacent niches in an environment or within the host once nutrients or space has been depleted [32], where it attaches again and restarts the cycle.

The implications of biofilms in treatment and outcomes are enormous, as they confer phenotypical resistance that required the use of new surgeries and prolonged treatments. It is therefore of utmost importance to avoid bacterial colonization of implants and thus avoid the appearance of infection. Moreover, the possibility of an interaction between biofilms, cells, and implanted biomaterials is also of great importance, as the reservoir in the tissue also needs to be removed to cure patients [34,35].

## 3. Conventional Prevention of Prosthetic Joint Infections

Conventional prevention of PJI includes all measures developed for preventing surgical site infections (SSIs) that have appeared in official guidelines and statements [36,37]. More specific measures for the prevention of PJI have also been published recently [38,39,40,41], and the importance of these measures was considered at the 2nd International Consensus Meeting at Philadelphia as a whole chapter in the General Assembly issues [42]. Factors increasing PJI risk can be grouped into three categories: preoperative, intraoperative, and postoperative [43]. Among the preoperative factors, some well-known ones are obesity, malnutrition, diabetes mellitus, smoking, skin decolonization before surgery, and nasal decolonization. Some important intraoperative factors are surgical-site hair removal, perioperative antibiotics whose use has been successful in reducing the risk of such infections by up to 80% [44,45], and perioperative antibiotic timing [13,46], surgical site skin decolonization, intraarticular irrigation by incorporating antiseptic substances, fibrinolytic agent use, wound closure, implant surface properties, and local antibiotic delivery, since, for instance, the use of a prosthesis cemented with antibiotic-loaded polymethyl-methacrylate cement has been proposed as a potentially useful method that diminishes the risk of PJI [47,48,49,50]. However, the use of antibiotic-loaded cements is not used in all patients so far, since its use has shown a high variability between cohorts, which is translated as a problem when comparing results [51] and requires the employment of specific heat-tolerant antibiotics. Among postoperative factors, some authors consider the typical temporal patterns of C-reactive protein, erythrocyte sedimentation rate test, interleukin 6, and D-Dimer in the early postoperative period [43].

However, even taking all those risk factors into account, there are still several patients who develop PJI after surgery. Several strategies have been devised to avoid this kind of infection.

## 4. Local Preventive Antibiotic-Based Strategies

During prosthetic implantation, the bone and surrounding tissue must be irrigated; in addition, after implantation, the periprosthetic tissue may be left damaged, avascular, or even, necrotic. These events inherent to surgery locally reduce the concentration of the antibiotic systemically administrated and make it necessary to use a local antibiotic approach with a period of action of hours or days.

On the other hand, the foreign body reaction after the implantation gives rise to an interstitial milieu or a *locus minus resistentiae*, which is an immunosuppressed fibro-inflammatory zone [52]. This zone is a relatively inaccessible environment for the immune response due to the absence of normal blood supply to the periprosthetic tissue [53], which impairs the ability of lymphocytes, antibodies, and certain antibiotics to properly reach the implant surface and thus prevent and fight infection via the systemic route. For this reason, any prosthesis would be susceptible to be infected not only during the perioperative period but also throughout its whole lifetime [54]. Therefore, a local antibiotic approach with an active period of months or years is required.

The ideal antibiotic-loaded titanium alloy surface modification would require two components: a titanium alloy component and an antibiotic component. The ideal titanium alloy surface modification must not compromise its good corrosion resistance, high strength, low weight, its Young’s modulus of elasticity [55], or non-cytotoxicity. In addition, this titanium alloy surface should be a selective surface able to impair the bacterial adhesion and to favor bone tissue integration [56]. The ideal antibiotic to be loaded should be a broad-spectrum drug based on local prevalence of antibiotic resistance with no adverse local or systemic effects. Further, the ideal antibiotic-loaded titanium alloy should fulfil some market requirements such as an acceptable cost, wide availability, and be easy to manufacture and overcome regulatory issues [57].

**Figure 1:**
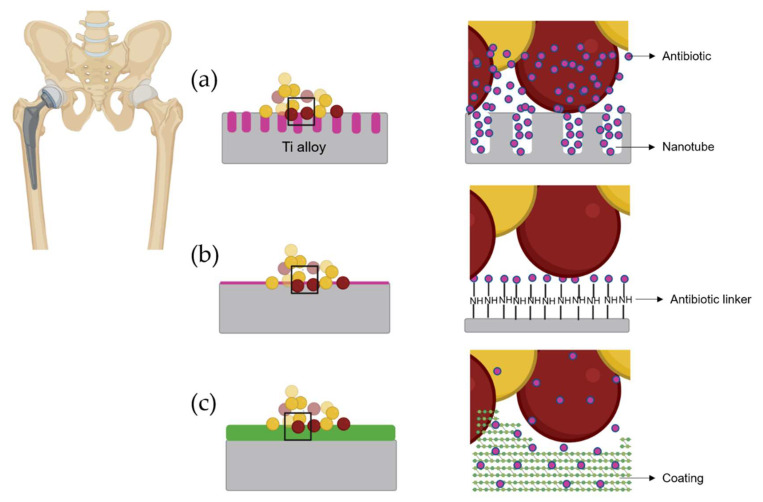
Different local antibiotic therapy strategies. (**a**) Antibiotic-loaded nanotubes. (**b**) Antibiotic covalently bound to titanium alloy. (**c**) Antibiotic-loaded coating. Yellow represents live bacteria. Red represents dead bacteria.

The local prevention approach can be classified into two types according to the mechanism of action: passive and active modifications. Passive modifications are surface coatings that endow biomaterial with antibacterial (anti-adherent, bacteriostatic, and/or bactericide) properties without releasing any compound that is responsible for these properties. The active modifications do endow biomaterials with antibacterial properties through a compound released from the material. These active modifications are divided into two groups: active surfaces and coatings. The most recent antibiotic-loaded surface modifications of titanium alloys are illustrated in Figure 1.

### 4.1. Active Titanium Surfaces Loaded with Antibiotics

The active titanium surfaces loaded with antibiotics can be divided into two categories: nanostructured surfaces and surfaces with covalently bound antibiotics.

The most representative nanostructured titanium surface approaches are summarized in Table 1. This strategy mainly consists of growing nanoscopic carriers made of the bulk alloy and loading them with at least one antibiotic. The most widely used nanostructure is the nanotube, a hollow cylinder without one of its circular faces. Nanotubes can be manufactured using different methods such as sol–gel synthesis, template-assisted synthesis, hydrothermal synthesis, and electrical anodization [58]. Among them, an exponential trend of the use of hydrothermal synthesis and electrical anodization can be observed over last two decades due to their multiple applications [59]. The hydrothermal synthesis modifies the crystallinity of the titanium precursor [60] and allows incorporating other chemical elements into the titanium nanotubes, which enhances their photoelectrochemical [1] properties [59] and confers interesting environmental applications involved, for instance, in the recalcitrant organic pollutant degradation [61]. However, between the two, the most versatile and used in the field of Biomaterials is electrical anodization due to its easy use and thrift.

This nanostructure allows its loading using different methods, mainly simplified lyophilization or soaking.

Bacterial and cellular adhesion are complex processes arising from the interaction between surface properties, biological factors, and environmental conditions. A recent systematic review concludes that there are three reasons why the relationship between surface topography and bacterial attachment can give rise to contradictory results: (i) roughness cannot be the sole descriptor of surface topography; (ii) topographical effects are influenced by the effects of other physicochemical factors, such as surface chemistry; and (iii) different anti-adherent mechanisms may take place at different topographical scales: nanoscale and microscale [62]. The last reason can be also applied to cell attachment. Some authors assert that titanium nanotubes increase the bacterial attachment but have excellent biocompatibility properties because of their enhanced protein interaction (including adsorption and conformation) what improves cellular adhesion and tissue growth [63]. Other authors, by contrast, assert that titanium nanostructures themselves can prevent [64] or reduce bacterial adhesion [65,66] or even biofilm development [67], and also promote cell adhesion and proliferation on the alloy [66,68]. Furthermore, nanotubes composition could be involved in part of these abilities. Thus, for instance, the incorporation of fluorine would be responsible for an anti-adherent ability [65], whilst the additional incorporation of phosphorus would be responsible for better osseointegration [69].

The nanotube diameter is pivotal for the release profile [70]; that is, the larger the diameter, the faster the release. Most of the nanotube-based approaches offer a constant antibiotic release for a few hours after surgery. As a result, this type of approach only guarantees local antibiotic with an active period of hours. The main antibiotic used for loading nanotubes are gentamicin [71,72,73] and vancomycin [74,75] in monotherapy since only few studies have used them in combination [76,77]. Gentamicin is a broad spectrum antibiotic effective against both Gram-positive and Gram-negative bacteria which has a great chemical stability since it remains stable at 4 °C for 30 days and at 23 °C for 7 days [78], and a great thermal stability due to this antibiotic retain its activity even after autoclaving [79]. For its part, vancomycin is a narrow spectrum antibiotic effective against Gram-positive bacteria, the main type of bacteria related to PJIs, and has a reduced chemical stability due to its the concomitant crystalline thermal degradation at physiologic condition [80], which can cause up to a 40% decrease in its activity in 3 weeks [81].

Antibiotics covalently bound to titanium surfaces is another type of active titanium surfaces with antibiotics (Table 2). The main techniques for covalently bound of antibiotics onto titanium surfaces involve the covalent attachment of end-functionalized polymers incorporating an appropriate anchor, e.g., silane anchor, catechol anchor, and phosphor-based anchor [82]. To date, numerous antibiotics have been employed using this strategy such as daptomycin [83], ciprofloxacin [84], doxycycline [85], vancomycin [86], enoxacin [87], bacitracin [88], a new antibiotic such as SPI031 [89], and even antifungals such as caspofungin [86].

### 4.2. Coating Loaded with Antibiotic for Titanium Alloys

Some of the most relevant coatings loaded with antibiotics described over the last 10 years are summarized in Table 3. In this period, strategies have focused on the design of coatings instead of nanostructures and the covalent binding of antibiotics. This reorientation of local antibiotic therapies may be justified by the huge versatility the coatings offer and their compatibility with not only titanium alloys, but also with almost any material from which a biomedical implant may be made.

Different approaches of deposition of antibiotic-loaded coatings such as sol–gel, covalent immobilization, spraying, electrophoretic, polyelectrolyte, and dip coating have been used on titanium surfaces [117]. Most of the coatings described are degradable over time and are composed of synthetic or natural polymers. The antibiotic release from these degradable coatings depends on their degradation or hydrolysis and the loaded antibiotic quantity depends on both the chemical composition of the coating and the chemical structure and chemical properties of the antibiotic used. The antibiotics that have been loaded onto these coatings are vancomycin [91,96,97,101,105,110,116], aminoglycosides (mainly gentamicin [93,99,100,106,109,113] and tobramycin), tetracyclines (especially doxycycline [98,103] and tetracycline) [102], cephalexin [111], moxifloxacin [112,118], and mixtures of antibiotics such as vancomycin plus tigecycline [108]. Further studies have demonstrated that antifungals, such as fluconazole and anidulafungin, loaded in a coating are effective to prevent *C. albicans* infection both in vitro [114] and in vivo [115].

The most commonly used synthetic polymers are poly (lactic-co-glycolic acid) (PLGA) (polycaprolactone/polyvinyl alcohol), poly (ethylene glycol-propylene sulphide), and poly-D,L-lactide. Most have been approved by the Food Drug Administration due to their biodegradability and biocompatibility in light of a vast number of recently reviewed studies [119,120]. New strategies based on the use of inorganic [92] and organo-inorganic sol–gels have recently emerged. Some of these organo-inorganic sol–gels have been shown to degrade into non-cytotoxic monomers [112], promote osteoblast proliferation [121], and can even prevent clotting [118]. The most representative natural polymers are based on the use of polysaccharides, e.g., chitosan and hyaluronic acid, and proteins, e.g., silk fibroin and collagen, whose use as drug delivery systems has been recently reviewed [122]. One of these coatings made of natural compounds, an antibiotic-loaded autologous blood glue [113], has attracted attention due to its enormous biocompatibility. This autologous blood glue is composed of a mixture of thrombin, platelet-rich plasma, and bone marrow aspirate and could therefore be loaded with gentamicin and become an antibacterial glue [113]. Several studies have evaluated the antibacterial efficacy of hybrid coatings made of biodegradable polymer and non-biodegradable material. Among them, it is important to consider gentamicin-loaded PLGA and hydroxyapatite, which improve the osteointegration of bone surrounding the implant [99]; vancomycin-loaded gelatin and mesoporous silica nanoparticles, which can carry antibiotic more efficiently [105]; and more complex coatings composed of agarose and nanocrystalline apatite for improved osseointegration, and with mesoporous silica nanoparticles loaded with cephalexin and vascular endothelial growth factor, able to promote vascularization surrounding the implant [123]. Hydroxyapatite coatings favor osteosynthesis [94,107] and prevent the development of fibrous tissue [124] surrounding the implant.

There are two marketed products based on the antibiotic-loaded degradable coating for titanium implants: gentamicin poly (D, L-lactide) (PLLA) coating, and a fast-resorbable hydrogel coating composed of covalently linked hyaluronan and PLLA. Gentamicin PLLA coating is based on a fully resorbable PLLA matrix loaded with gentamicin sulphate which releases 80% of its antibiotic load within the first 48 h [125]. Gentamicin PLLA coating is named PROtect Coating and is only marketed coating Expert Tibial Nail (DePuy Synthes, Bettlach, Switzerland). Though its use is limited to tibial intramedullary nail, it might be theoretically used on any titanium implant. In the first prospective study, Fuchs et al. [126] demonstrated that none of the 19 patients with closed or open tibial fractures who completed the 6-month follow-up showed implant-related infections. Similar results were obtained by Metsemakers et al. [98] in a single-center case series, where they demonstrated again its capacity of preventing implant-related infections in 16 patients with complex open tibia fracture and revision cases after an 18-month follow-up, but they also reported 25% of patients showed a nonunion, and 6.25% of them was a revision case. Finally, the most recent and largest study performed by Schmidmaier et al. [127] in a multicenter study analyzed the outcome of 99 patients with fresh open or closed tibial fractures or undergoing nonunion revision surgery. After an 18-month follow-up, deep SSI or osteomyelitis was only noted in 7.2% of patients after fresh fracture and in 7.7% of patients after revision surgery.

Fast-resorbable hydrogel coating is composed of covalently linked hyaluronan and PLLA and is marketed as Defensive Antibacterial Coating (DAC) (Novagenit Srl, Mezzolombardo, Italy). DAC is the first antimicrobial hydrogel specifically designed to avoid implant-related infections in orthopaedic surgery and trauma, dentistry, and maxillofacial surgery [128,129]. Its antimicrobial ability is due to the hyaluronic-based compounds that reduce microbial adhesion and biofilm formation of both bacteria and yeasts [130]. Moreover, the DAC has demonstrated itself to be capable of entrapping several antibacterial agents at concentrations ranging from 2–10%, released locally for up to 72 h [128]. The safety and efficacy of DAC have been demonstrated by using rabbit models that revealed the capacity of the vancomycin-loaded hydrogel to prevent implant-related infection [131,132]. In a further rabbit model, vancomycin-loaded DAC-coated implants showed no detrimental effects on the bone healing and implant osteointegration [133]. In the first large multicenter randomized prospective clinical trial reported by Romanò et al. [134], a total of 380 patients were included. The patients were randomly dived into two groups which received an implant with the DAC intraoperatively loaded with antibiotics (gentamicin, vancomycin, or vancomycin plus meropenem) or without the coating (control group). Overall, 96.5% of patients were available at a mean follow-up of 14.5 ± 5.5 months. Eleven SSIs were diagnosed in the control group (6%), whilst only one was observed in the treatment group (0.6%). Any patient from the treatment group showed no local or systemic side effects related to or detectable interference with implant osteointegration. In another multicenter prospective study performed by Malizos et al. [135], 256 patients undergoing osteosynthesis surgery for a closed fracture were randomly assigned to receive the DAC loaded with antibiotics (gentamicin, vancomycin, or vancomycin plus meropenem) or to a control group without coating. Six SSIs (4.6%) were observed in the control group compared with none (0%) in the treatment group after a mean follow-up of 18.1 ± 4.5 months. As in the previous study, any patient from the treatment group showed no local or systemic side effects related to or detectable interference with implant osseointegration.

Trentinaglia et al. [136] have recently described an algorithm to calculate the cost-effectiveness of different antibacterial coating strategies applied to joint prostheses, considering both direct and indirect hospital costs. According to their model, an antibacterial coating able to decrease post-surgical infection by 80%, at a cost per patient of EUR 600, would reduce hospital costs by EUR 200 per patient if routinely applied in a population that would theoretically show an expected PJI rate of 2% [137]. At a European level, considering that approximately 2.2 million joint arthroplasties are performed per year, they speculate that a year of delay in the routine use of this kind of coating would result in 35,200 PJI cases per year with associated annual costs of approximately EUR 440 million per year [137].

### 4.3. The Antibiotic of Choice for Local Antibiotic-Based Therapy

The use of almost any antibiotic in clinical practice is always followed by the development of resistant organisms, and the case of antibiotic-loaded titanium surfaces is not an exception. Antimicrobial resistance is the result of three major factors: (1) the increasing frequency of antimicrobial-resistant phenotypes among microbes resulting from selective pressure exerted by the widespread use of antimicrobials; (2) globalization, which favors the rapid spread of pathogens worldwide; and (3) improper use of antibiotics [138].

The antibiotic of choice for local antibiotic-based therapy should ideally be a broad-spectrum antibiotic that is the least allergenic possible and with no local adverse effects or cytotoxicity; furthermore, these antibiotics should not interfere with osseointegration or be essential for the treatment of PJI [56]. Most of the local antibiotics of choice are broad-spectrum antibiotics used in monotherapy, concretely gentamycin, tobramycin, and vancomycin. To date, there is no antibiotic that is evolution-proof [139,140], as any antibiotic monotherapy is associated with the emergence of antibiotic resistance to that particular antibiotic. This has been described previously, for instance, when a gentamicin-loaded spacer was used in a two-stage replacement which favored the emergence of gentamicin-resistant *S. aureus* [141] and *S. epidermidis* [142]. Therefore, the best prophylactic therapy should be based on the use of at least two antibiotics from different antibiotic families, as a handful of studies have done [76,94,108,143]. The microorganisms tested are staphylococci and, to a lesser extent, Gram-negative bacteria, such as *E. coli* and *P. aeruginosa*. Given the incidence of PJI (up to 40%) [144], Gram-negative bacteria should always be prevented by the local antibiotic approach.

## 5. Local Preventive Heavy Metals-Based Strategies

The increasing prevalence of antibiotic resistance among bacteria resulting in the selective pressure which the widespread use of antibiotics exerts on them, the globalization, and the inadequate use of antibiotics in many different settings [138] threaten to completely impede the development of an ideal preventive antibiotic therapy for any type of infection. Given this scenario, new non-antibiotic antimicrobials are gaining increasing importance in the field of PJI prevention strategies (Table 4).

**Figure 2:**
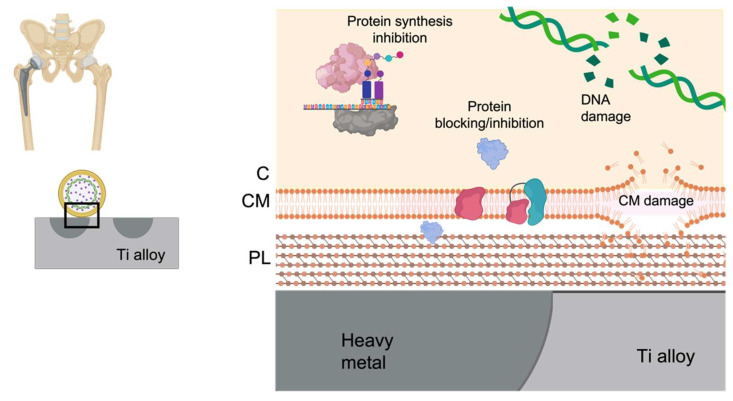
Main antibacterial mechanisms of heavy metals. PL: peptidoglycan layer. CM: cytoplasmatic membrane. C: cytoplasm.

Metals have been used by the Persians, Phoenicians, Greeks, Romans, and Egyptians for their antimicrobial properties for thousands of years [145,146]. Despite the fact that the exact mechanism involved in their broad-spread antibacterial mechanism remains unknown, metals show a higher number of unspecific targets within the bacteria, unlike the antibiotic, which is directly related to a reduced not null emergence of metal resistance. These targets are attacked by metallic cations and/or reactive oxygen species generated by both cations and by metallic oxide [147]. Thereby, the main antibacterial mechanisms of metals that show an antibacterial effect per se can be grouped into four categories: (outer and/or cytoplasmatic) membrane damage, protein blocking/inactivation, protein synthesis blocking, and DNA damage [145] (Figure 2). Different strategies have incorporated heavy metals into titanium surfaces. The main heavy metals used to provide titanium alloys with antimicrobial capacity are silver, copper, and gallium. The type of surface modification used to incorporate the metal on the titanium surfaces are mainly metallurgical addition, co-sputtering, ion implantation, and coatings.

Regarding the use of these metallic-based titanium alloy surface modifications in patients, it is noteworthy that there are no comparative or prospective studies and only retrospective cases of series have been published. Only silver has been proven in humans and has shown low infection risk in clinical studies. There are two technologies marketed nowadays for incorporated silver into titanium alloys: anodization and galvanic deposition. Titanium alloy prostheses with silver incorporated by anodizing is marketed under the name Agluna^®^ (Accentus Medical, Oxfordshire, UK). Anodizing gives rise to the formation of 5 μm diameter circular tanks in the surface of the prosthesis, containing an amorphous titania species where the bulk of the ionic silver is stored. Silver galvanic deposition into titanium alloy prostheses is marketed under the name MUTARS^®^ (tumor system components; Implantcast GmbH, Buxtehude, Germany). Its technology consists of a 15 ± 5 μm-thick silver coating deposited by galvanic deposition on a 200 nm layer of gold that acts as a carrier and bonding layer to the prosthesis. Recently, Deng et al. [173] have pointed out that some factors might underestimate the real anti-infective effect of silver-modified prostheses in clinical studies. First, most of indications published vouch for the use of this type of prosthesis in immunocompromised patients, those with musculoskeletal tumors [174,175,176,177] and/or with a previous PJI [175,176,178], and patients who are themselves more vulnerable to developing PJI [179]. Second, the antibiotherapy is usually administered to all patients, whether or not they carry silver-modified prostheses.

The use of heavy metals for PJI prevention may just be getting started, thus new promising metallic candidates with antimicrobial capacity are yet to be employed. This is the case for metals such as nickel [180,181], cerium [182], selenium [183,184], cesium [185], yttrium [186], palladium [187,188], or superparamagnetic Fe NPs [189].

## 6. Limitations Associated with Local PJI Prevention

Despite all the potential benefits offered by local prevention strategies for prosthetic joint infections, each has several limitations associated with its use. The advantages and disadvantages related to each preventive approach of PJI are summarized in Table 5.

Titanium nanotubular surfaces have at least five limitations. Firstly, the low drug concentration resulting from sustained release in a non-bacteria environment consumes antibiotic reserves and increases the possibility of developing drug-resistant bacteria in the vicinity of the implant [58]. Therefore, the ideal antibiotic release of a nanotube-based approach should terminate after the infection is eliminated until the next stimulus [58]. This perspective would require the use of self-responsive nanotubes able to release antibiotics before different infection scenarios. Secondly, any metallic implant in the human body degrades due to at least four fundamental phenomena: leaching, wear, corrosion, as well as the phenomenon resulting from the synergy between the latter two, tribocorrosion. Wear studies about the properties of nanostructured titanium surfaces are scarce, and it is known that wear proprieties of nanotubular titanium surfaces have to be hypothetically different as non-nanostructured surface and these nanostructures can be damaged during the prosthesis implantation; nanostructures pulled from the surface could act as debris, able to cause an aseptic loosening [190]. Nanotube fabrication increases the surface area and hence the corrodible area. Corrosion studies of Ti-6Al-4V implants in patients showed that the detection of elevated levels of titanium and normal levels of aluminum and vanadium (relative to a control group without loosening) in the serum or urine of wearers of a prosthesis made of this alloy was associated with the existence of aseptic loosening [191,192,193,194]. Thirdly, nothing is known about the repercussions that this corrosion may have on the useful life of the implant or its osseointegration. Fourthly, the current load methods require the employment of specific equipment (vacuum ovens, agitators, etc.) and long loading times, which make it impossible to load them in the operating theatre for the time being. Fifthly, this approach has no clinical trials to support its widespread use in humans and marketing.

Regarding antibiotics covalently bound to titanium surfaces, there are also important limitations associated with this approach. Unlike nanostructured surface, the antibiotics covalently bound to surfaces are not released into the milieu, and thus can only exert their action on bacteria in direct contact with the modified surface. There is no information about the exact durability of their protection or the hypothetical effect of the release of chemically modified antibiotic on the target bacteria and its role on the emergence of antibiotic resistance. The chemical reaction needed for obtaining these surfaces makes the intra-operative antibiotic load impossible. Finally, there are no clinical trials to back up their use in humans.

Antibiotic-loaded coatings also show limitations. The main limitation is the incomplete protection of the implant, since the intramedullary component of the prosthesis and some modular components (e.g., the acetabular component and the polyethylene insert) cannot be coated. Therefore, an area of susceptibility will exist, where a bacterial infection could proliferate. There is absence of knowledge about the long-term effect that the product resulting from its degradation could exert on the useful life of the implant, its osseointegration, or even, the patient coagulation profile. Although it is the only approach with clinical trials, few antibiotics loaded in such coatings have been used so far.

Heavy metals into titanium surfaces are also associated with some limitations. First, the price of these modified implants is high because they are indicated for a very low number of specific patients [173]. Second, the heavy metals are linked to both local and systemic toxicity. The main side effects of local toxicity are the immunosuppressive effect [195] and the poor or impaired osteointegration that has been reported by both in vitro [196] and in vivo [197] studies. The main systemic side effect related to a titanium alloy surface modified with heavy metals has been described for silver. Argyria, a disease caused by a high silver concentration in the human body, has been reported in up to 23% patients that underwent megaendoprostheses for infection or resection of malignant tumors [198]. In this cohort, no neurological, renal, or hepatic symptoms of silver poisoning were found, and neither a relationship between argyria and the size of the implant or levels of serum silver [198]. Therefore, more studies about the silver intoxication caused by silver-coated implants need to be performed.

Therefore, toxicity is the first concern pertaining to these modifications. With a silver coating, the elevated silver concentration in the blood or in organs has been proven by Gosheger et al. [34], while there were no detectable clinical side effects in this study. The silver ion concentration was lower than the reported harmful concentration, which could be an explanation. Argyria, a disease caused by physiologic silver cation overload, was reported in nearly 22% patients who have received silver-coated prostheses [67]. Therefore, the release of silver ions to the human body after implantation of silver-coated prostheses should be investigated [52]. Impaired osteointegration, which is a special concern for arthroplasty, was generally tested in in vitro co-culture models [68].

Other limitations include the selection of antimicrobial compound. For preventive use, narrow-spectrum antibiotics that cover most potential pathogens are recommended for chemoprophylaxis [36,37]. However, because some antibiotics, such as beta-lactams, can degrade with different factors, such as time or temperature, more stable antibiotics (for example, vancomycin, gentamicin, quinolones) are chosen in many studies. Another important problem not directly related to the biomaterial is the increasing burden of infections caused by antibiotic-resistant microorganisms [8]. The problem of antimicrobial resistance is currently considered one of the most important menaces facing modern medicine [199]. The recent appearance of multidrug-resistant microorganisms has become an extremely important problem with implications for all aspects of medical practice. In orthopaedic surgery, the increasing number of multidrug-resistant organisms, especially Gram-negative organisms, has been described in PJI [8]. This type of infections caused by these microorganisms implies a poor outcome in many cases [200,201,202]. Even silver or copper as heavy metals representants can give rise to heavy metal-resistant Gram-negative bacteria (mainly *E. coli* and *P. aeruginosa*) [203,204], one of the bacterial groups related with PJI that is increasing its incidence [205].

In this scenario, the selection of the antimicrobials necessary to prevent PJI infections should consider the existence of multidrug-resistant bacteria [206], which emphasizes the need to select a mixture of at least two antibiotics for preventing PJIs or even using more than one of the preventive approaches described here, e.g., an antibiotic-loaded and heavy metal-dopped surface modification, but also drives the search for new strategies based on the use of iodine-doped titanium alloys [207], antimicrobial peptides [208], and bacteriophages [209,210,211], among others.

## 7. Conclusions

Research into the development of locally antibiotic therapy approaches is broad and varied, though this review could mark the beginning of a promising journey towards the development of prostheses capable of complete PJI prevention. Despite the numerous preclinical studies that have been conducted, such as those using in vivo models, the move from bench to bedside continues to be hindered by at least two factors, including the low incidence of PJIs and the costs of clinical trials needed to demonstrate the efficacy of these approaches in human beings; indeed, these costs are so high that only large pharmaceutical companies can afford such an investment. These factors may be responsible for the fact that existing multicenter prospective clinical trials are poorly well-structured and often show contradictory or inconclusive results [212]. Thus, the only way patients can benefit from these promising approaches is by improving collaboration between governments, regulatory agencies, industry leaders, and health care payers [213].

Our review highlights that a trend from the antibiotic-loaded surface modifications of the bulk material to the biodegradable antibiotic-load coating can be observed since only two types of these coatings have come to be used in humans. Among heavy metals, silver-modified titanium surfaces are supported by numerous in vitro studies and clinical trials, though other metals such as copper or gallium might stand up as potential future candidates. Furthermore, there is no uniform way of evaluating the efficacy of such approaches. For that, we consider that at least cytotoxicity and cell proliferation should be evaluated in vitro, and that all be tested by using in vivo models. Due to the increasingly threatening emergence of antibiotic resistance, it would therefore be recommendable to use at least two antibiotics or heavy metals for functionalizing the titanium surfaces or antimicrobial substances whose antibacterial mechanisms do not lead to the development of resistant bacterial mutants. Finally, any of the PJI prevention approaches reviewed here are exempt of limitations, many of which should be elucidated by specifically designed studies.

## Figures and Tables

**Table 1:** Some of the most relevant studies based on titanium nanotubes loaded with antibiotics.

Year	Type of Surface Modification	Bacteria Evaluated	Bacterial State	Cytotoxicity (%)	Level Study	Cell Lines/Animal Used In Vivo	Reference
2014	Gentamicin-loaded nanotubes with different diameters	SA, SE	Biofilm	ND	In vitro	hBMMS cells	[71]
2016	Chitosan-coated gentamicin-loaded nanotubes	SA	Planktonic	20	In vitro	MG-63 osteoblasts	[72]
2017	Gentamicin-loaded nanotubes made with anodization	SA	Biofilm	ND	In vivo	‒/rabbit	[73]
2018	Chitosan-hyaluronic acid-coated vancomycin-loaded nanotubes	SA	Planktonic/Biofilm	0	In vitro/in vivo	Primary osteoblasts/rat	[74]
	Vancomycin-loaded micro-patterning	MRSA	Biofilm	ND	In vivo	‒/rabbit	[75]
	Gentamicin and/or vancomycin F-dopped nanotubes	SA, SE, EC	Planktonic	ND	In vitro	‒/‒	[66]
2019	Gentamicin plus vancomycin F- and P-dopped bottle-shaped nanotubes	SA	Biofilm	0	In vitro/in vivo	MC3T3-E1 osteoblasts/rabbit	[76]

Abbreviation: SA: *S. aureus*; SE: *S. epidermidis*, EC: *E. coli*; MRSA: Methicillin-resistant *S. aureus*; ND: Not determined. hBMMS cells: Human marrow-derived mesenchymal stem cells.

**Table 2:** Some of the most relevant studies based on antibiotic covalently bound to titanium surfaces.

Year	Antibiotic Covalently Bound	Bacteria Evaluated	Bacterial State	Cytotoxicity (%)	Level Study	Cell Lines/Animal Used In Vivo	Reference
2010	Daptomycin	SA	Biofilm	ND	In vitro	‒/‒	[90]
2014	Doxycycline	‒	‒	0− <40	In vitro/in vivo	MC3T3-E1 osteoblasts/rabbit	[85]
2015	Ciprofloxacin	PA	Biofilm	0	In vitro/in vivo	NIH3T3 fibroblasts/mouse	[84]
2016	Vancomycin/caspofungin	SA, CA	Biofilm	0	In vitro/in vivo	hME cells/rat	[86]
	SPI031	SA, PA	Biofilm	0	In vitro/in vivo	hBMMS cells, hME cells/mouse	[89]
	Enoxacin	MRSA, SE, EC	Planktonic, Biofilm	0	In vitro/in vivo	hBMMS cells/rat	[87]
2017	Bacitracin	SA	Biofilm	ND	In vivo	‒/rat	[88]

Abbreviations: SA: *S. aureus*; SE: *S. epidermidis*, EC: *E. coli*; PA: *P. aeruginosa*; MRSA: methicillin-resistant *S. aureus*; ND: Not determined. hBMMS cells: Human marrow-derived mesenchymal stem cells. hME cells: human microvascular endothelial cells.

**Table 3:** Some of the most relevant studies based on antibiotic loaded coating for titanium implants.

Year	Type of Coating	Evaluated Bacteria	Bacterial State	Cytotoxicity (%)	Level Study	Cell Lines/Animal Used In Vivo	Reference
2010	Vancomycin-loaded PMMA	SE	Biofilm	ND	In vitro	‒/‒	[91]
	Inorganic sol–gel with Polymyxin B covalently bound	EC	Planktonic	ND	In vitro	‒/‒	[92]
	Gentamicin-loaded polyelectrolyte multilayer	SA	Planktonic, Biofilm	0–80	In vitro/in vivo	MC3T3-E1 osteoblasts/rabbit	[93]
2014	Rifampicin and fosfomycin-loaded Hydroxyapatite coating	MSSA, MRSA	Biofilm	ND	In vivo	‒/rabbit	[94]
	Ciprofloxacin-loaded chitosan-nanoparticles coating	SA	Planktonic	<30	In vitro	MG63 osteoblast-like cells	[95]
	Chitosan–vancomycin composite coatings	SA	Planktonic	0	In vitro	MG63 osteoblast-like cells	[96]
	Vancomycin-loaded PLGA-coating	SA	Planktonic/Biofilm	0	In vitro	MC3T3-E1 osteoblasts/rabbit	[97]
2015	Doxycycline-loaded polymer-lipid encapsulation matrix coating	MSSA, MRSA	Planktonic, Biofilm	ND	In vitro/in vivo	‒/mouse	[98]
2015	PLGA-gentamicin-hydroxyapatite-coating	SA, SE	Planktonic, Biofilm	ND	In vitro/in vivo	‒/rabbit	[99]
2016	Gentamicin-derivates coating	SA	Biofilm	ND	In vivo	‒/rats	[100]
2016	Vancomycin-loaded phosphatidyl-choline	SA	Biofilm	ND	In vivo	‒/rabbit	[101]
2016	Tetracycline loaded chitosan-gelatin nanosphere coating	SA	Biofilm	>90	In vitro/in vivo	MC3T3-E1 osteoblasts/rabbit	[102]
2017	Doxycycline-loaded coaxial PCL-PVA nanofiber coating	SA	Biofilm	ND	In vivo	‒/rat	[103]
	Tobramycin-loaded PDLLA coating	SA	Biofilm	ND	In vivo	‒/rabbit	[1]
2018	Vancomycin-loaded mesoporous bioglass-PLGA coating	SA	Planktonic, Biofilm	0	In vitro	hBMMS cells	[104]
	Vancomycin-loaded mesoporous silica nanoparticles-containing gelatin coating	SA	Biofilm	0	In vitro	hBMMS cells	[105]
2019	Gentamicin-loaded polyelectrolyte multilayer	SA, SE	Planktonic, Biofilm	<5	In vitro/in vivo	MC3T3-E1 osteoblast/rats	[106]
	Tobramycin-loaded hydroxyapatite coating	SA	Planktonic, Biofilm	ND	In vitro/in vivo	Endothelial cells, primary osteoblasts/rabbit	[107]
	Vancomycin plus tigecycline-loaded PEG-PPS coating	SA	Biofilm	ND	In vivo	‒/mouse	[108]
	Gentamicin-loaded calcium phosphate-based coating	SA	Biofilm	ND	In vivo	‒/rat	[109]
	Vancomycin-loaded polymethacrylate coating	SA	Planktonic/Biofilm	ND	In vitro/in vivo	‒/mouse	[110]
2020	Cephalexin- and VEGF-loaded agarose-nanocrystalline apatite coating	SA	Planktonic	0	In vitro	MC3T3-E1 osteoblast	[111]
	Moxifloxacin-loaded organic-inorganic sol–gel	SA, SE, EC	Planktonic, Biofilm	0	In vitro/in vivo	MC3T3-E1 osteoblasts/mouse	[112]
	Gentamicin loaded autologous blood glue	PA	Planktonic, Biofilm	0	In vitro	hBMMS cells	[113]
	Fluconazole/anidulafungin-loaded organic-inorganic sol–gel	CA, CP	Planktonic, Biofilm	0	In vitro	MC3T3-E1 osteoblasts	[114]
	Anidulafungin-loaded organic-inorganic sol–gel	CA	Biofilm	-	In vivo	‒/mouse	[115]
	Vancomycin-loaded starch coating	SA	Planktonic	ND	In vitro	‒/‒	[116]

Abbreviations: PLGA: poly(lactic-co-glycolic acid); PCL-PVA: polycaprolactone/polyvinyl alcohol; PEG-PPS: poly(ethylene glycol-bl-propylene sulfide); PDLLA: poly (D, L-lactide); SA: *S. aureus*; SE: *S. epidermidis*, EC: *E. coli*; PA: *P. aeruginosa*; MRSA: methicillin-resistant *S. aureus;* MSSA: Methicillin-susceptible *S. aureus*; CA: *Candida albicans*; CP: *Candida parapsilosis*; ND: Not determined. hBMMS cells: human bone marrow mesenchymal stem cells.

**Table 4:** Some of the most relevant studies based on heavy metals incorporation for titanium implants.

Year	Type of Surface Modification	Incorporated Metal	Metal Incorporation	Bacteria Evaluated	Bacterial State	Cytotoxicity (%)	Level Study	Cell Lines/Animal Used In Vivo	Reference
2009	Metallurgical addition	Cu	Forge	SA, EC	Planktonic/biofilm	Ctyocompatible	In vitro/ in vivo	V79 cell line/rabbits	[148]
2010	Co-sputtering	Cu-Mn-O, Ag-Mn-O	ternary and quaternary oxides	SA, SE	Planktonic	-	In vitro	-	[149]
	Single step silver plasma immersion ion implantation	Ag	Nanoparticles	SA, EC	Planktonic	Cytocompatible	In vitro	MG63 human osteoblast-like cells	[150]
2011	TiO_2_-chitosan/heparin coating	Ag	Nanoparticles	SA	Biofilm	-	In vivo	-	[151]
	Hydroxyapatite coating	Ag	Nanoparticles	EC	Planktonic	-	In vitro	-	[152]
2013	Metallurgical addition	Cu	Powder metallurgy	SA, EC	Planktonic	-	In vitro	-	[153]
	Titanium nanotubular	Ag	Nanoparticle loading	SA, EC	Planktonic	-	In vitro	-	[154]
	Polydopamine-modified alloy surface	Ag	Silver inonic inmobilization	EC	Planktonic	-	In vitro	-	[155]
	Poly(ethylene glycol diacrylate)-co-acrylic acid coating	Ag	Nanoparticles	SA, EC, PA	Planktonic	Cytocompatible	In vitro	MG63 human osteoblast-like cells	[156]
2014	Metallurgical addition	Cu	Powder metallurgy	SA, EC	Planktonic	-	In vitro	-	[157]
	Metallurgical addition	Cu	Casting with post-treatment	SA, EC	Planktonic	Cytocompatible	In vitro	L929 cell line	[158]
	BMP-2/heparinchitosan-hydroxyapatite coating	Ag	Nanoparticles	SE, EC	Planktonic	Cytocompatible	In vitro	MC3T3-E1 cells, BMS cells	[159]
	Aminosilanized titanium alloy	Ag	Nanoparticles	SA	Planktonic	-	In vitro	-	[160]
2016	Metallurgical addition	Ag	Sintering	SA	Planktonic	-	In vitro	-	[161]
2017	Metallurgical addition	Ag	Sintering, casting, casting with appropiate post-treatment w/o surface tretament	SA	Planktonic	Cytocompatible	In vitro	MC3T3-E1 cells	[162]
2018	Metallurgical addition	Cu	Powder metallurgy	SA, EC	Planktonic	Cytocompatible	In vitro	HeLa cells	[163]
	Metallurgical addition	Ag	Spark plasma sintering and acid etching	SA	Planktonic	Cytocompatible	In vitro	MC3T3-E1 cells	[164]
	Metallurgical addition	Cu	Casting with post-treatment	SA	Planktonic	-	In vitro	-	[165]
2019	Metallurgical addition	Cu	Sintering	SA	Biofilm	-	In vivo	-	[166]
	Metallurgical addition	Ga	Powder metallurgy	MRSA	Planktonic/biofilm	Cytocompatible	In vitro	ATCC CRL-11372 and ATCC HTB-96	[167]
2020	Metallurgical addition	Cu	Microwave sintering	SA, EC	Planktonic	-	In vitro	-	[168]
	Metallurgical addition	Cu	Powder metallurgy	EC	Planktonic	-	In vitro	-	[169]
	Metallurgical addition	Ag	Casting with appropiate post-treatment w/o surface tretament	SA	Planktonic	Cytocompatible	In vitro	MC3T3-E1 cells	[170]
2021	Metallurgical addition	Cu	As-cast	SA	Biofilm	-	In vitro/in vivo	Mouse	[171]
	Metallurgical addition	Cu	As-cast	MRSA	Planktonic/biofilm	Cytocompatible	In vitro/in vivo	MC3T3-E1 cells/rat	[172]

Abbreviations: BMP-2: bone morphology protein-2; BMS: bone marrow stromal cells.

**Table 5:** Some of the most important advantages and disadvantages related to each preventive approach of PJI.

Preventive Approach of PJI	Advantages	Disadvantages
Antibiotic-based strategies		
Nanostructured titanium surfaces	Possibility of increasing the osteointegration of the titanium surfaces	Reduced durability of antibiotic protection
		Unknown biomechanical stability
	Loaded antibiotic can act against both bacteria directly adhered on the titanium surface and bacteria near but not in contact with it	Unknown effects on the useful life of the implant, osteointegration, and coagulation profile
		Impossibility of intra-operative antibiotic load No clinical trials to support their use
Antibiotics covalently bound to titanium surfaces	Long durability of antibiotic protection, up to months or years	Loaded antibiotic can only act against bacteria directly adhered on the titanium surface
		Unknown durability of antibiotic protection
		Impossibility of intra-operative antibiotic load
		No clinical trials to support their use
Coatings loaded with antibiotic for titanium alloys	Possibility of increasing the osteointegration of the titanium surfaces	Incomplete surface protection
	Loaded antibiotic can act against both bacteria directly adhered on the titanium surface and bacteria near but not in contact with it	Unknown effects on the useful life of the implant, osteointegration, and coagulation profile
	Possibility of intra-operative antibiotic load	
	Clinical trials to support their use	Clinical trials that support their use has been carried out with few antibiotics
Heavy metals-based strategies	Broad spectrum antimicrobial effect (beyond antibacterial effect)	Local and systemic toxicity supported by clinical trials
	Loaded metals can act against both microorganisms directly adhered on the titanium surface and those near but not in contact with it	
	Long durability	
	Clinical trials to support their use	

## Data Availability

The data presented in this study are available on request from the corresponding author. Some data are not publicly available since some articles are not open access.
